# Initiation of reticular and spider veins, incompetent perforantes and varicose veins in the saphenous vein network of the rat

**DOI:** 10.1038/s41598-020-71982-z

**Published:** 2020-09-21

**Authors:** Bernadett Bettina Patai, Gabriella Dornyei, Anna-Maria Tokes, Judit Reka Hetthessy, Alexander Fees, Gyorgy L. Nadasy

**Affiliations:** 1 Department of Traumatology, Military Hospital, Budapest, Hungary; 2 Department of Morphology and Physiology, Health Sciences Faculty, Semmelweis University, Budapest, Hungary; 3 Molecular Oncology Research Group, 2nd Department of Pathology, Semmelweis University, Budapest, Hungary; 4 Department of Orthopedics, Medical Faculty, Semmelweis University, Budapest, Hungary; 5 Department of Physiology, Semmelweis University, Tuzolto Str 37-47, 1094 Budapest, Hungary

**Keywords:** Cardiovascular biology, Angiogenesis, Cardiovascular biology

## Abstract

In an attempt to induce experimental varicosity, reverse perforant vein development was initiated in the rat leg by applying a chronic (14 and 32 weeks) partial stricture on the main branch of the deep femoral vein. At surfacing of the incompetent perforantes, typical reticular vein plaques and spider veins were identified by video-microscopy and quantitative histology. Deep vein blood was channeled by them into the saphenous vein system, the extra flow deforming these vessels, causing local dilations and broken course, even undulations of larger branches.

## Introduction

The human leg varicosity disease still does not have a viable animal model. In a recent publication we reported that after chronic partial occlusion of the saphenous vein main branch of the rat, a rich collateral network developed, with morphological similarity to the reticular veins of the initial phase of the human leg varicosity disease. Expression of the contractile protein smooth muscle actin as well as of elastin was limited in their walls in the first 4–8 weeks, while accelerated cell division and monocytic infiltration further weakened their mechanical firmness. Subjecting them to chronic gravitational load (maintenance in tilted tube-cages) resulted in local dilations and undulating courses resembling varicosity^1^.

With the advent and wide application of advanced sensitive ultrasonographic and laser-illumination techniques, as well as by further improving skills of physical examination, many phlebologists are now of the opinion that in most leg varicosities, *incompetent perforantes* can be identified that “feed” the pathologically deformed superficial network^2–11^. While normal perforant veins connect the superficial and deep venous systems of the leg from the superficial to the deep direction only and reverse flow is made impossible due to the normal functioning of valves in them, pathologic, incompetent perforantes will not prevent reverse flow and muscle pump activity may drive deep venous blood not upward, toward the common femoral vein, but in the reverse direction, toward the superficial (saphenous) vein system. To mimic that situation, in a new series of experiments, we applied a chronic, local narrowing stricture on the main branch of the deep femoral vein of rats, just before its confluence with the saphenous vein. The resulting collateral venous network was studied by injection of methylene blue stained saline into the deep femoral vein and observing its appearance in the veins on the surface of the thigh and leg muscles. Their structure was examined after further microsurgical exploration by video-microscopy and on histological sections.

## Results

**Figure 1:**
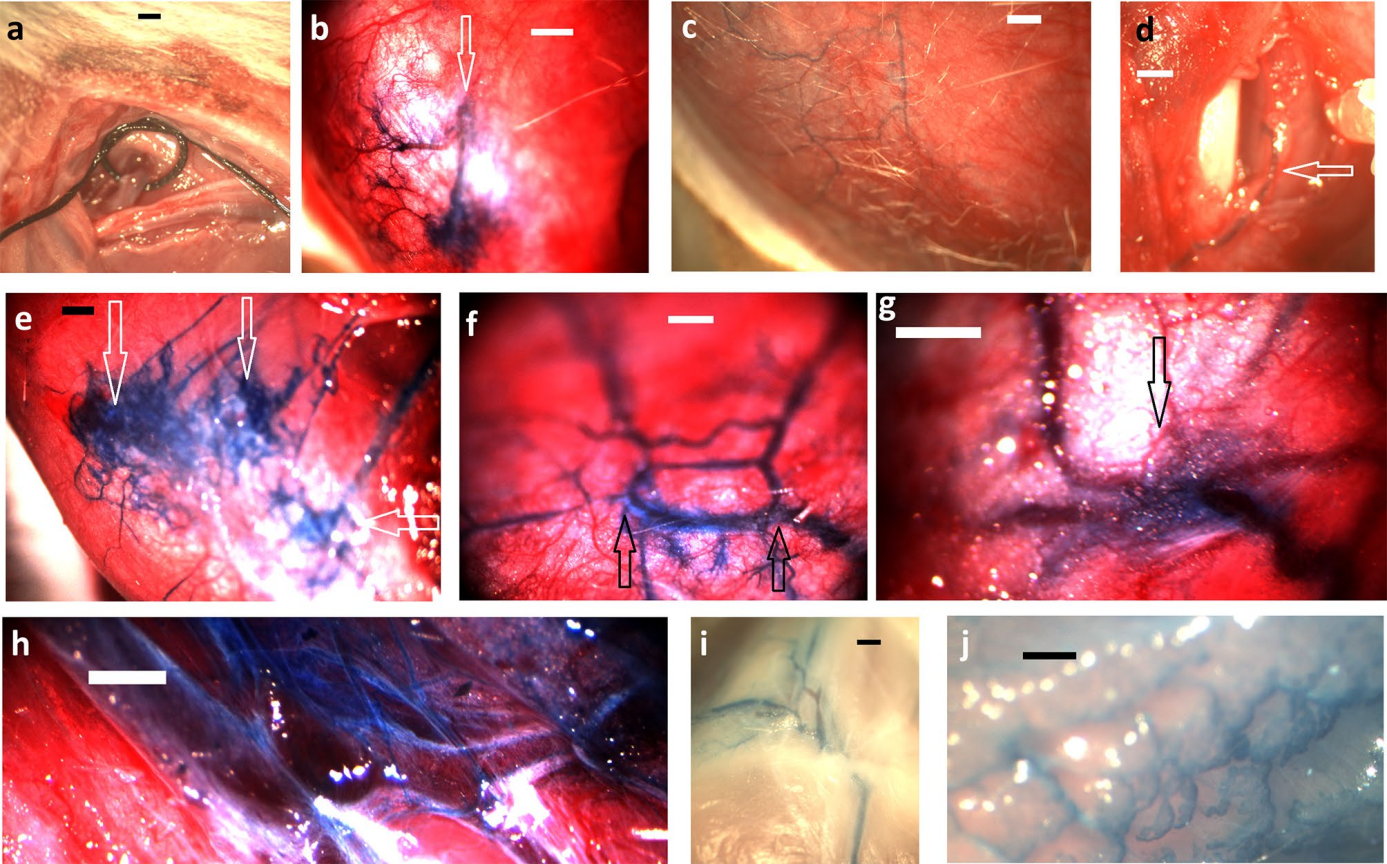
Chronic partial occlusion of the deep femoral vein. Methylene blue injection into the deep femoral vein. (**a**) The isolated main branch of the deep femoral vein before its stricture. (**b**) The dye surfacing at occlusion side into reticular vein plaques. (**c**) Normal venules and small veins filling from the deep veins through the microcirculation. (**d**) Incompetent perforans microprepared following its course. (**e**–**g**) Reticular vein plaques and spider veins at surfacing of incompetent perforantes (arrows). (**h**–**j**) Varicous larger veins at occlusion side. Scale bars for (**a**,**b**,**e**,**i**) 1,000 µm, (**c**,**d**,**f**,**g**,**h**,**j**) 500 µm.

Fourteen and 32 weeks after partial occlusion of the deep femoral vein, at reoperation, it was found that scar tissue fully closed the deep femoral veins at stricture site. However, sufficient collaterals developed to prevent any venous tissue flow disturbance. Part of such collaterals were real *reverse perforant veins* (Fig. 1d) able to empty their blood into reticular vein plaques at the muscle surface (Fig. 1b). Surprisingly, there was some connection between deep and superficial venous systems at the control, non-strictured side, too (Fig. 1c), but it was through the normal microcirculation only. At occlusion side, in each case we could identify typical pathological reticular vein masses and spider veins (Fig. 1b,e,f,g), while such structures were practically nonexistent at the control, non-occluded side (Table 1, *p* < 0.01 with the χ2 probe). Reticular vein blood flow was channeled into larger saphenous vein branches, however, this extra load induced morphological deformations on the latter. *Local dilations, broken course and undulating course* were typical in the affected saphenous vein network (Fig. 1h,i,j).

**Table 1:** Comparison of superficial venous networks with and without chronic deep femoral vein stricture.

	Control side	Strictured side	
Number of reticular vein plaques^a^	2	20	*p* < 0.01
Number of spider veins^a^	0	9	*p* < 0.01
Deep vein blood surfaces at pressure (mmHg)^b^	11 ± 2.5	19.1 ± 3.2	n.s.
SMA staining on cross section^c^	56.5%	66.4%	*p* < 0.01
Elastin (RF) staining on cross section^d^	58.2%	70.6%	*p* < 0.01
Collagen staining on cross section^d^	54.5%	43.2%	*p* < 0.01

^a^Pooled data, 11 animals, 14 weeks deep vein stricture, comparison with the Khi^2^ probe.

^b^9 animals, 32 weeks deep vein stricture, comparison with one-way anova.

^c^Pooled data, 9 animals, 25 veins, 32 weeks deep vein stricture, high intensity staining measured as low blue, comparison of the frequency curves with the Khi^2^ probe.

^d^Pooled data, 9 animals, 25 veins, 32 weeks deep vein stricture, high intensity staining measured as low green,

comparison of the frequency curves with the Khi^2^ probe.

When the dye was injected into the deep femoral vein in a controlled, slow manner, appearance on the surface was at somewhat lower pressures at the occluded than at the control side, however, this difference did not reach the level of statistical significance (Table 1).

**Figure 2:**
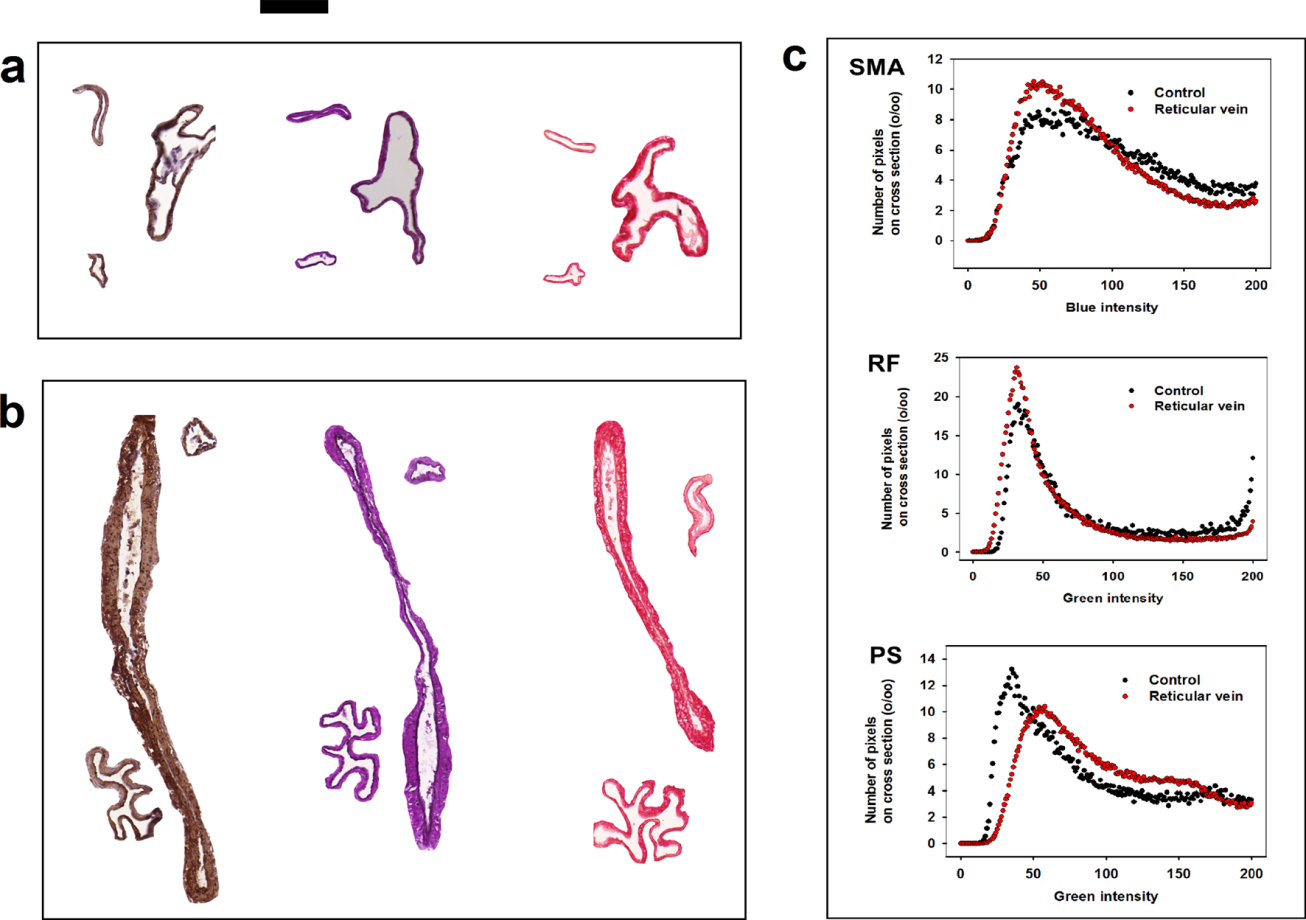
Histological sections of normal small veins, side branches of the saphenous vein at contralateral side (**a**) and morphologically dilated veins found in reticular vein plaques (**b**). Stained with (left to right) smooth muscle actin immunochemistry (brown, DAB), resorcin fuchsin (magenta, elastica) and PicroSirius (collagen, red). Scale bar, 100 µm. (**c**) Quantitative evaluation of wall tissue components in normal side branches and in reticular veins. Density histograms for blue (suppressed by SMA DAB brown) and green (suppressed by RF’s magenta elastica and PS’s red collagen) colors. Relative frequency of staining intensity is given in thousands of venous wall cross sections.

Histological investigations after 32 weeks of occlusion found in reticular vein plaques widely dilated vessels, their walls contained of elastin and contractile protein in sufficient amount, but, interestingly, less collagen (Fig. 2 and Table 1).

## Discussion

**Figure 3:**
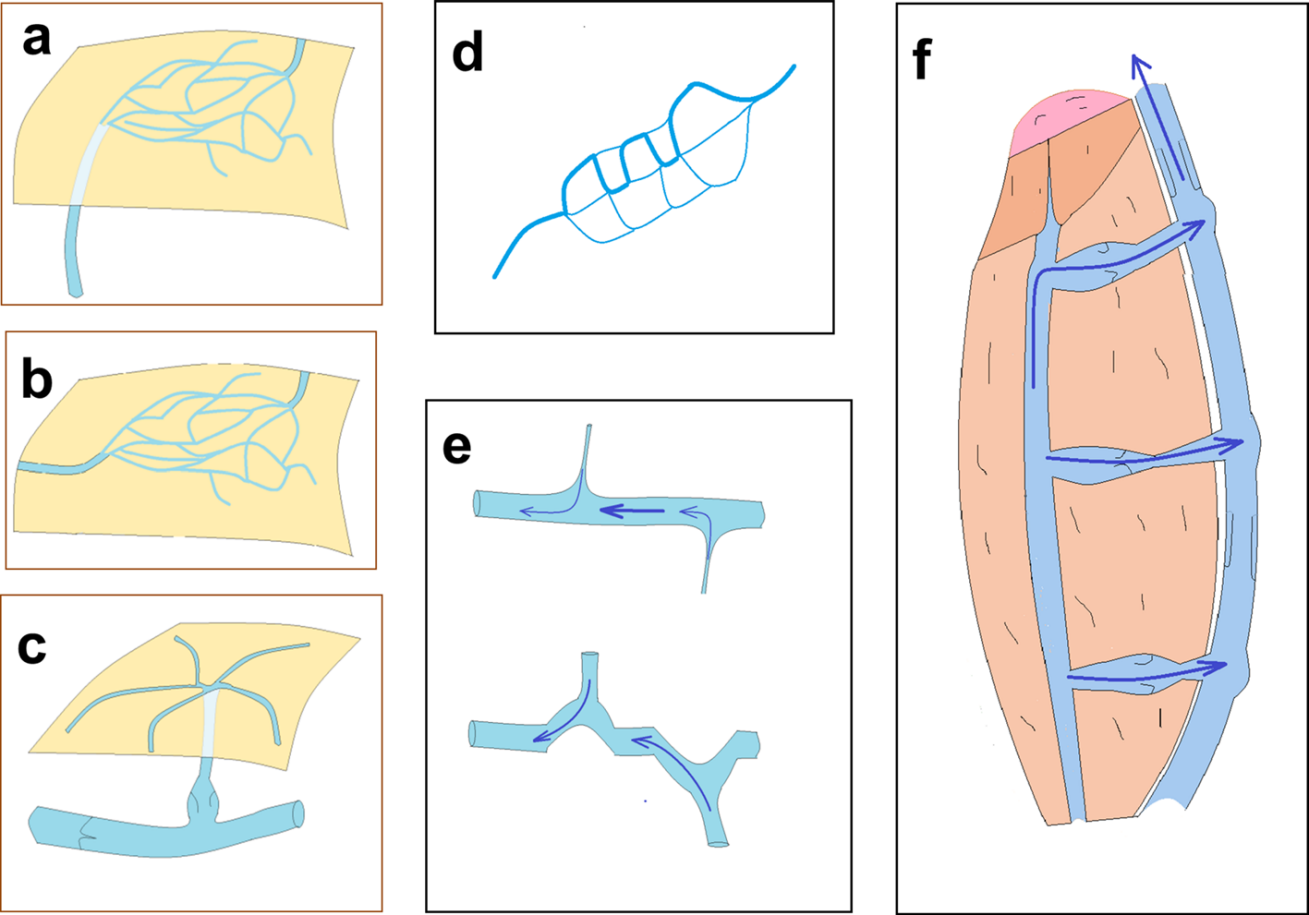
Explanation of the pathomechanism of varicous morphologies. (**a**) Reticular vein plaque at surfacing of an incompetent perforant vein. (**b**) Reticular vein plaque fed by a more or less superficial “feeder vein”. (**c**) Spider veins at surfacing of an incompetent perforant vein. (**d**) Undulating course as a result of morphological enlargement of certain branches in a reticular vein network. (**e**) Undulating course of a larger saphenous vein branch as a result of morphological lateral “drag” exerted by a side branch with substantially elevated flow. (**f**) Suggested mechanism how inhomogenous contraction of antigravitational muscles can induce flow obstacle accompanied with high transmural pressures in perforant veins. Strong isometric contraction (deep brown) practically closes upward flow in deep vein main branch. Periodic or lighter contractions in muscle mass below (light brown) then pump blood in the reverse direction, toward the superficial network and produce incompetent perforantes.

According to our observations obtained in these animal experiments, *reticular vein* plaques will develop where there is a flow obstacle in a larger vein and venous blood should find its way through several small tributaries connected parallel and in series, occasionally having no valves^12^ to inhibit reverse flow. Blood then will percolate through the system of this reticular vein plaque, being collected at its other side and channeled into a larger branch now in the common anterograde direction of the superficial network. The chronically high flow^13^ induces then the pathologic remodeling of the reticular venous mass^1^. Reticular vein masses can be fed either directly by an incompetent perforans (Fig. 1b,d,e,f,g, explained in Fig. 3a) or by a more or less superficial “feeding vein” (Fig. 3b). If somewhat larger veins have formed the original superficial tributaries of a perforant vein, *spider veins* can develop as a result of their filling from the reverse direction (Fig. 1f,g explained by Fig. 3c). Deep femoral vein blood will be channeled into the saphenous vein system, causing morphological flow dilation and *pathological deformation* of its branches as the result of the deviation of the main branch’s course toward the side branches with enormously elevated blood flows (Fig. 1h,i, explained in Fig. 3e). Another possibility for the development of the convoluted course in the superficial venous system is the morphological strengthening of those branches and segments in the reticular vein masses, that have higher bloods flows (Fig. 1j, explained in Fig. 3d).

In contrast to the 4–8 week collaterals we had studied in our earlier work after saphenous vein partial obstruction^1^ these reticular veins did not contain less than normal amount of elastin and smooth muscle contractile protein. The 32 weeks was enough for the expression of the intra-and extracellular force-bearing elements However, they still had less dense collagen and being much wider than normal veins, even that amount of force-bearing proteins could not be sufficient to resist gravitation (Fig. 2 and Table 1).

Clinicians will be aware of the fact, that the mechanism suggested here is resembling of the secondary saphenous varicosity observed as a complication of deep vein obstruction due to a chronic thrombotic process^14^. To obstruct deep venous blood flow, however, no thrombosis or stricturing sutures are needed. Our suggestion is that during the development of the frequent human low extremity varicosity disease, inhomogenous contraction of the leg and thigh antigravitational muscles can induce a similar situation: strong, sustained contraction can close the upper part of deep femoral veins similarly to our partial stricture, while a less strong or intermittent contraction below that level pumps blood toward the surface (Fig. 3f). This results in the development of incompetent perforantes as an indirect effect of gravitation.

The morphological similarity with certain pathological features of the human leg varicosity disease is obvious, there is a reason to think that many histological and cellular processes will show a similarity with different phases of it. Several pathological mechanisms present in the human disease can be studied under convenient experimental conditions, new means of therapy can be first tested without any human risk. However, we have to admit that the exact human situation, with a partial genetic adaptation to the upright body position^15^ will not be fully modeled.

## Conclusion

We have found that *morphological flow remodeling* is the most important mechanism in the background of pathological deformations of small and large veins in the development of the leg varicosity disease. By the partial occlusion of the deep femoral vein in the rat we have found a technique to produce varicous networks. This should be yet further developed to yield a quantitatively and qualitatively sound translational model of this important human disease.

## Methods

The deep femoral vein was partially strictured in anesthetized male Wistar rats (Pentobarbital 45 mg/kg body weight, i.p.) in a microsurgical intervention (Fig. 1a): a multifilament thread was tied around a 450 µm (26G) needle forming a narrowing loop of that diameter. No complications were observed, animals were moving freely, no edema, discoloration or movement disorder developed. All interventions applied during the study conform to the guidelines from Directive 2010/63/EU of the European Parliament on the protection of animals used for scientific purposes, NIH guidelines and local regulations. The program has been accepted by the Animal Care Committee of the Semmelweis University and Hungarian authorities (PEI 001/801-2/2015, PE/EA/1430-7/2018).

In the first series (11 animals, 409 ± 14 gr at start of study) animals were reanesthetized after 14 weeks (body weight 612 ± 30 gr), the deep femoral vein was microcannulated and Krebs–Ringer solution stained with methylene blue was injected into its lumen. The appearance of the dye was observed on the surface of the thigh and leg muscles (with skin removed). Stained superficial veins were photographed either immediately or after some additional microsurgical preparation better to show their structure. Points of surfacing of reversely conducting perforant venous branches were identified, their courses were carefully microprepared to demonstrate the direct reverse connection between deep and superficial veins. The contralateral leg served as control. In the second series (9 animals, 433 ± 10 gr) similar technics of deep vein narrowing and observation were applied but more time, 32 weeks were left for the collaterals to develop. In reanesthesia (weight, 608 ± 25 gr), animals were bled, the lower body separated, its blood vessels washed with heparinized Krebs–Ringer solution through the abdominal aorta. Then the deep femoral veins were cannulated as above, and methylene-blue stained Krebs–Ringer solution was injected into them in a controlled manner, 0.1 ml/min while pressure was continuously measured. Flow was elevated up to 0.5 ml/min if needed to ensure slow (< 5 mmHg/min) pressure elevation up to 25 mmHg. Pressures at which the methylene blue appeared first on the surface of the skinned thigh or leg were recorded. Infusion was continued until full development of the collateral pathways. Histological samples were collected containing the superficial veins with filling from the deep femoral veins, directly from areas containing the reticular veins and spider veins (occlusion side) and the normal, orthogradely conducting saphenous side branches at the contralateral non-strictured side. Histological sections, 5 µm thick, were stained with resorcin-fuchsin (RF, for elastica), Picrio-Sirius (PS, for collagen). Immuno-histochemistry for the contractile protein, smooth muscle actin (SMA) was also performed (R&D Systems Inc primary rabbit antibodies, secondary goat antibodies, visualized with the DAB technique). Histological stainings were made with an automatic histological device (Ventana Benchmark XT Immune-Automat System), controls and occlusion sides were stained in the same series. Pictures were scanned (3D Histech Pannoramic250 Scanner) photographed at 20 × magnification using the Pannoramic Viewer software (pixel size 1.5 µm). Venous cross sections of pathologic and control veins were cut from the pictures. The Image J software (National Institute of Health) was used to compute the color intensity histograms for blue, which was suppressed by the SMA’s DAB brown and green, which was suppressed by RF’s magenta-colored elastica and by the PS’s red stained collagen.

## Data Availability

Original data are available upon request.
